# Synaptic Reshaping and Neuronal Outcomes in the Temporal Lobe Epilepsy

**DOI:** 10.3390/ijms22083860

**Published:** 2021-04-08

**Authors:** Elisa Ren, Giulia Curia

**Affiliations:** 1 Department of Biomedical, Metabolic and Neural Sciences, University of Modena and Reggio Emilia, 41125 Modena, Italy; elisa.ren@unimore.it; 2 Center for Neuroscience and Neurotechnology, University of Modena and Reggio Emilia, 41125 Modena, Italy

**Keywords:** temporal lobe epilepsy, Human, animal model, *Status epilepticus*, pilocarpine, kainic acid, kindling, chemical synapses, GABA receptors, glutamate receptors, gap junctions

## Abstract

Temporal lobe epilepsy (TLE) is one of the most common types of focal epilepsy, characterized by recurrent spontaneous seizures originating in the temporal lobe(s), with mesial TLE (mTLE) as the worst form of TLE, often associated with hippocampal sclerosis. Abnormal epileptiform discharges are the result, among others, of altered cell-to-cell communication in both chemical and electrical transmissions. Current knowledge about the neurobiology of TLE in human patients emerges from pathological studies of biopsy specimens isolated from the epileptogenic zone or, in a few more recent investigations, from living subjects using positron emission tomography (PET). To overcome limitations related to the use of human tissue, animal models are of great help as they allow the selection of homogeneous samples still presenting a more various scenario of the epileptic syndrome, the presence of a comparable control group, and the availability of a greater amount of tissue for in vitro/ex vivo investigations. This review provides an overview of the structural and functional alterations of synaptic connections in the brain of TLE/mTLE patients and animal models.

## 1. Introduction

Epilepsy is a widespread and worldwide chronic brain disorder affecting approximately 1% of the world’s population, with important implications in the quality of life of sufferers [1]. Among the wide spectrum of epilepsy disorders, temporal lobe epilepsy (TLE), often refractory to antiepileptic drugs (AEDs; [2]), has emerged as the most common type referred for epilepsy surgery [3]. Spontaneous recurrent seizures (SRSs) originate within one or both temporal lobes and can secondarily generalize, causing variable levels of impairments in learning, memory, and mood, which, together with the SRSs, characterize epileptic syndromes [4,5,6]. Mesial TLE (mTLE) is the most severe form of TLE and is often associated with hippocampal sclerosis and, in fewer cases, with amygdala enlargement [7,8]. Morphometric magnetic resonance imaging (MRI) evaluation of both hippocampal and parahippocampal areas in mTLE patients has confirmed extensive atrophy in the anterior portion of the mesial temporal lobe, in particular in the hippocampal head and body and in the entorhinal cortex, with impairment of entorhinal-hippocampal circuits [9,10]. From a histopathological point of view, hippocampal sclerosis, considered an important biomarker of mTLE, is characterized by a conspicuous neuronal loss in the *Cornu Ammonis* (CA) regions and reactive gliosis. These neuropathological features have been investigated to evaluate different types of mTLE based on the classification of different degrees of sclerotic severity (e.g., ILAE (International League Against Epilepsy) classification system [11], or on Wyler’s grading system [12]). TLE patients with no sclerotic hippocampus may present extra-hippocampal volume alterations [13] and are commonly characterized by various degrees of reactive astrogliosis [11]. Additionally, changes in the neuronal density, granule cell dispersion, and circuit reorganization, for example, due to mossy fibers sprouting, blood-brain barrier integrity impairment, and neuroinflammation, represent other important hallmarks of TLE syndrome [14,15].

**Figure 1:**
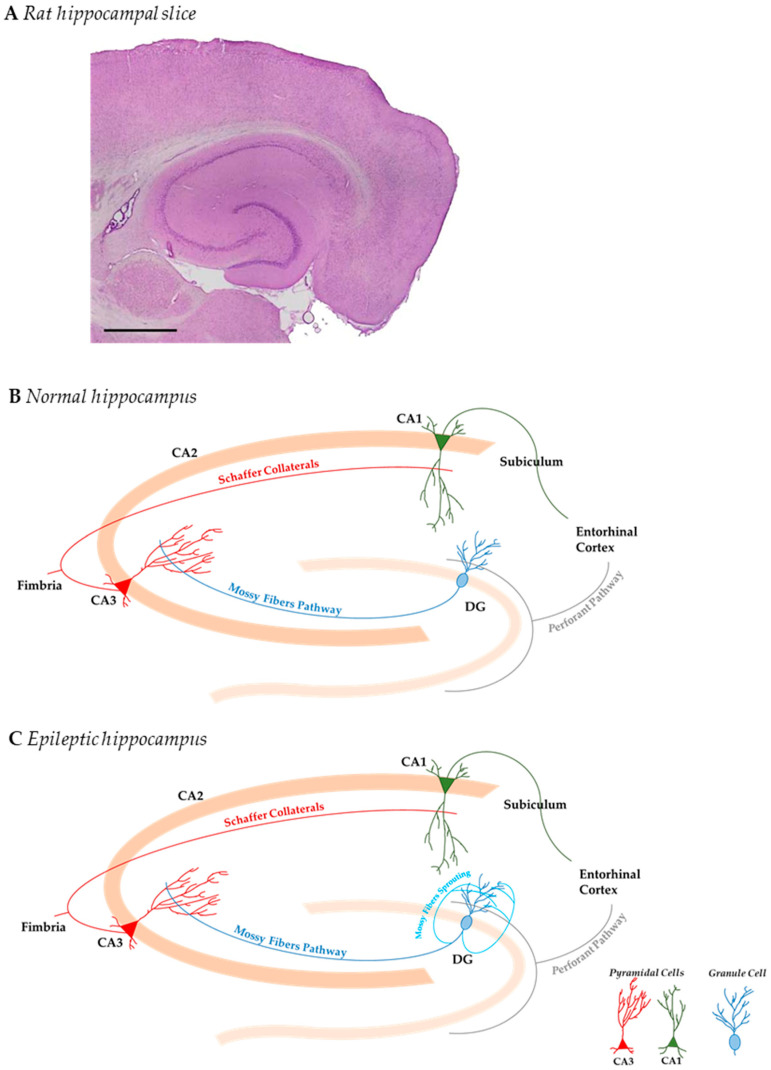
Graphical representation of hippocampal anatomy and connectivity in normal and epileptic temporal lobe epilepsy (TLE) brain. (**A**) Hematoxylin and eosin stained the hippocampal structure in a transverse section from an adult male rat brain (Scale bar: 1 cm). (**B**) Graphical representation of hippocampal functional circuits shows the anatomical subfields of the dentate gyrus (DG) and *Cornu Ammonis* (CA) areas. These hippocampal subfields are functionally inter-connected through granule mossy fibers (light blue) and Schaffer Collaterals (red) and receive major cortical input from the entorhinal cortex (perforant pathway). (**C**) The epileptic hippocampus undergoes mossy fiber sprouting, which consists of a circuit reorganization of granular cell axons (dark blue) into the dentate inner molecular layer, contributing to aberrant excitation of the hippocampal network.

Anatomical subfields of the dentate gyrus, CA3, CA2, and CA1, are functionally interconnected (Figure 1) and connected to extra-hippocampal structures such as the medial and lateral entorhinal cortexes [16]. Normal hippocampal circuitry is at the bases of storage and recall of memories [17], while alterations to it are found in TLE [18,19,20].

TLE, in common with other epileptic syndromes, is traditionally thought to be the result of altered neuronal excitability, and modifications in cell-to-cell communication in both chemical and electrical transmissions are among the leading causes [21]. Ictal events in the epileptic hippocampus are commonly manifested by an imbalance of chemical excitatory and inhibitory signaling that are mainly governed by glutamate and γ-aminobutyric acid (GABA) neurotransmitters, respectively. Furthermore, altered expression and function of gap junctions suggest the possible involvement of uncoupled electrical synapses among the mechanisms underlying the pathophysiology of TLE as well [21].

Current knowledge about the neurobiology of epilepsy in humans mainly emerges from surgical resections from patients with pharmacoresistant TLE who underwent temporal lobectomy as the only therapeutic option available [22]. The resulting specimens represent a rare chance to study the composition of the epileptic tissue, specifically with regard to the quantification and functioning of both synapses and neurotransmitter receptors [23]. Indeed, these isolated specimens preserve the native functional neuronal circuitry, representing an accurate substrate for the investigation of mechanisms involved in epileptic activities [24]. More recent investigations could be performed on living subjects thanks to forefront technologies such as positron emission tomography (PET). With the application of imaging tracer, PET allows presurgical clinical study of the epileptogenic foci and the observation of receptor density in the living human brains of people with refractory focal epilepsy [25,26,27]. In all cases, sampling bias due to the selection of subjects that may not be representative of the whole spectrum of TLE scenarios and the lack of proper control groups, including healthy subjects, can create various limitations in the study of TLE using human tissue. In this regard, animal models are of great help as they allow (i) a more uniform experimental group still showing high subject-to-subject variability, representing a wide variety of epileptic scenarios, from mild to very severe syndrome, induced by the same brain insult [13], (ii) the availability of a greater amount of tissue for in vitro/ex vivo investigations which is not “contaminated” by AEDs treatments, and (iii) the presence of a comparable control group.

*Status epilepticus* (SE) is one of the most widely used brain insults to trigger limbic epileptogenesis in animal models resulting in TLE. SE can be induced by intraperitoneal or intracranial (focal) injection (i.e., hippocampal, cerebroventricular striatal, and amygdala) of chemoconvulsants, such as pilocarpine or kainic acid (KA) [28,29], or by repetitive electrical stimulation [30]. In particular, pilocarpine induces the activation of the cholinergic system via M1 muscarinic acetylcholine receptor subtypes [31], with significant implications in the hippocampal CA1 and CA3 regions, the amygdala, piriform cortex, olfactory cortex, thalamus, and substantia nigra [32]. KA is a potent neuroexcitatory substance found in some red marine algae or seaweeds that activates ionotropic glutamate α-amino-3-hydroxy-5-methylisoxazole-4-propionic acid (AMPA) and KA receptors, leading to brain damage and limbic seizures [33]. Electrical stimulation to induce SE, known as the limbic kindling model [34], is applied by focal repeated electrical stimulation in the hippocampal area [30] or in the amygdala [35]; this model has also been used to study mood psychiatric disorders [35], often encountered in TLE human patients [36].

This review provides an overview of the structural and functional alterations of chemical and electrical synaptic connections in the brain from TLE/mTLE patients and animal models. An extensive search was conducted in the PubMed database. The search included studies up to 30 January 2021. The search terms used were: temporal lobe epilepsy, human, animal model, *Status epilepticus*, pilocarpine, kainic acid, kindling, chemical synapses, GABA receptors, glutamate receptors, gap junctions, positron emission tomography. The boolean operator AND was used to connect these descriptors to develop search strings. To be included, studies had to be: original research, published in a peer-reviewed journal, written in English, on human patients affected by TLE or animal models in which TLE was chronically established. Exclusion criteria were set as: studies missing control (non-epileptic) group, data on epileptogenesis or collected during the latent stage after brain insult for animal studies, reviews, conference abstracts, or not in English. Reviews are mentioned only in the introduction or for general information about receptor/subunits but not for data on subunit/receptor expression in TLE/mTLE versus non-epileptic tissue.

## 2. Chemical Synapses Features in Temporal Lobe Epilepsy

Chemical synapses are structured by the apposition of a presynaptic terminal and a post-synaptic membrane separated by a synaptic cleft. The communication between the pre- and post-synaptic entities is mostly mediated by glutamate in excitatory synapses and by GABA for inhibitory synapses, respectively. Glutamate and GABA propagate their action through the interaction with receptors protein embedded in the postsynaptic membrane consisting of ionotropic ligand-gated ion channels and metabotropic G-protein coupled receptors subfamilies. The coexistence of these receptors and their placement within the synaptic domain are critical for the fine control of neuronal networks, and the potential excitation-inhibition imbalance in the generation of seizures is commonly accepted as one of the major causes of epilepsy [37].

### 2.1. Glutamatergic Excitatory Synapses

In the vertebrate central nervous system (CNS), glutamatergic neuronal transmission is mediated by the activation of fast excitatory ionotropic receptors, classified as AMPA, N-methyl-D-aspartate (NMDA), and KA receptors, and metabotropic glutamate receptors (mGluRs). Glutamate plays important functions in the healthy brain since it oversees 80%–90% of all synapse connections [38]. It is predominantly found in neuronal cells [39], but it also acts as a gliotransmitter released from astrocytes, contributing to the fine regulation of the synaptic network [40,41]. Synaptic dysfunctions affecting the balance of glutamatergic transmission, with reference to altered expression of glutamate receptors and anomalous neurotransmitter uptake and turnover machinery, attract considerable attention to investigate the physiopathology of chronic epilepsy in tissue obtained from human sufferers [42,43].

#### 2.1.1. AMPA Receptors

AMPA receptors are tetrameric transmembrane proteins sustaining a cationic conductance and are composed of four subunits GluA1–GluA4 [44]. GluA1, 2, and 3 are highly expressed in the whole human CNS, in particular in the hippocampus, mainly within the CA1–CA3 region, in the granular cells of the dentate gyrus, as well as other brain areas such as the temporal lobe, amygdala, and thalamus [45]. AMPA receptors are expressed in the active zone and lateral plasma membrane in both the pre- and post-synaptic endings [46].

Researches involving human TLE specimens reveal a general increment in the mRNA and protein levels of AMPA receptors in the hippocampus, dentate gyrus, lateral amygdaloid nucleus, and the temporal neocortex [47,48,49] (Table 1). Compared to the brains of healthy volunteer individuals, the elevation of AMPA receptors has been recently confirmed in the epileptogenic area of the living human brain with refractory TLE using a novel PET radiotracer, [^11^C]K-2, with a higher affinity for GluA2 and GluA4 for in vivo detection [27]; despite the evidence for overall lower synaptic densities in the epileptogenic area of TLE suffers due to neuronal loss, the [^11^C]K-2 signal has been seen to be elevated in the epileptic focus, underling an increased density of AMPA receptors in the synapses of living cells and confirming an up-regulation of functional AMPA receptors, regardless single receptor subunits distinction, in the temporal neocortex of epileptic patients compared to post-mortem non-epileptic tissues [27].

In the human sclerotic hippocampus, GluA1 subunits seem to be specifically increased in the dentate gyrus, hilar mossy cells, and CA areas pyramidal neurons in comparison with postmortem non-epileptic tissues [48,50,51,52]. Interestingly, Babb and colleagues confirmed the increase in GluA1 protein in mossy fibers in the rat KA model of TLE [50], while downregulation of GluA1 protein was reported in hippocampal and temporal lobe tissues obtained from chronic epileptic rats in other two studies [46,53].

Moderately mRNA level increase in GluA2 was found in dentate granule cells of mTLE patients in contrast with non-epileptic autopsy cases (i.e., tumors, vascular abnormality) [48,52]. GluA2 expression in the hippocampus is instead controversial: Mathern observed a moderate increase in GluA2 mRNA level in the sclerotic hippocampus of mTLE patients compared to non-epileptic autopsy cases [52], Hamamoto’s team reported no difference in GluA2 gene expression in mTLE hippocampus specimens compared to those from healthy human autopsies [54], while other studies found a decrease in protein level in surgical specimens obtained from TLE refractory patients compared to those of postmortem autopsies [51] and in animal TLE models [46,53]. GluA2-containing AMPA receptors are believed to be impermeable to Ca^2+^; thus, a shift towards an elevation of GluA2-lacking receptors, for example, GluA1/GluA1 homomers over the most abundant GluA1/GluA2 heteromers, may favor increased intracellular Ca^2+^ content, cell death, and enhanced neuronal excitability with seizure spread [46,51]. Noteworthy, downregulation of GluA1 and GluA2 AMPA subunits were also specifically observed in different subsynaptic compartments of Shaffer collateral-CA1 synapse, possibly leading to long-term potentiation (LTP) alterations [46]. The elevated expression of AMPA receptors in the lateral amygdaloid nucleus (which is thought to be critically involved in the generation of interictal discharges in the epileptic brain) [47] was not confirmed in other amygdaloid nuclei. Jafarian and colleagues indicated no variations in AMPA receptors, specifically investigating GluA1 and GluA2 subunits within basolateral and centromedial nuclei in human mTLE tissues compared to control individuals with no known history of neuropsychiatry disorders [55]. More recently, gene expression of GluA2 subunit was observed to be upregulated in the amygdala of human mTLE cases compared to amygdala specimens from healthy human autopsies; however, details about specific expression in various nuclei were not provided, and GluA1 expression was not investigated [54].

Variable GluA3 subunit expression levels were found in the human mTLE dentate gyrus and Ammon’s horn region [48,52], while in epileptic rats, a significant reduction in protein concentration was identified in the hippocampus, but not in the temporal lobe [53].

Despite growing evidence about the presence of ionotropic AMPA receptor expression in glial cells, their physiological role in human astrocytes is less understood [56,57]. Human astrocytes collected from pharmacoresistant TLE patients showed no differences in the expression of mRNAs encoding glutamate receptor subunits of AMPA receptors with an intermediate Ca^2+^ permeability compared to non-epileptic human biopsies [57]. Each AMPA subunit can originate the flip and flop variants due to RNA alternative splicing, exhibiting different properties; for example, desensitization kinetics are slower in the presence of flip variants, therefore making the cells much prone to cytotoxic effects [58,59]. In this regard, an increased expression of the flip form of GluA1, but not of GluA2/4 subunits, is increased in astrocytes obtained from human mTLE with hippocampal sclerosis compared to TLEs characterized by lesions outside of the hippocampus [57].

#### 2.1.2. NMDA Receptors

NMDA receptors are composed of seven subunits GluN1, GluN2A–D, and GluN3A–B, organized into tetrameric receptor structures at both the pre-synaptic and post-synaptic terminals [60]. NMDA receptors have traditionally been considered to be diheteromeric, composed of sets of GluN1/GluN2 or GluN1/GluN3 pairs. However, the substantial overlap in the expression of all three subunits also permits the existence of triheteromeric NMDA receptors, composed of GluN1, GluN2, and GluN3 subunits [61]. These receptors present the voltage-dependent block by extracellular Mg^2+^ and high Ca^2+^ permeability [62]. Moreover, unlike the AMPA receptor, NMDA receptors present slower activation and desensitization mechanisms, mainly due to the GluN2 subunit [56], while GluN3-containing receptors have reduced affinity for Mg^2+^ and increased selective permeability for Ca^2+^ over Na^+^, making them highly Ca^2+^ permeable [63].

Tissue from human amygdalohippocampectomy and neocortical tissue from TLE patients shown variable levels of NMDA receptors density and gene expression [47,48,49,64] (Table 1); increased functional NMDA receptors have been observed in the dentate gyrus and has been associated with mossy fiber sprouting [64], while no changes in receptor density were evident in the human lateral amygdala [47]. Recently, using the tracer [^18^F]GE-179 for in vivo PET scanning, elevated NMDA channel activation was confirmed at least in one patient showing ictal electroencephalographic activity in the temporal lobe among patients suffering from different forms of focal epilepsy [26].

The expression of GluN1 subunits was observed as being reduced in the mTLE hippocampus [54], not confirming previous findings that reported no relevant modifications in GluN1 mRNA and protein level both in mTLE and TLE hippocampus of humans and animal models [52,53], along with a slight increase in tissues of the sclerotic dentate gyrus [48,52]. In the amygdala of mTLE patients compared to amygdala samples resected from an autopsy group not presenting neuropsychiatric symptoms, the expression of the GluN1 was observed significantly increased [54,65], but Jafarian and colleagues revealed no difference between the two experimental groups in the basolateral nucleus and central amygdaloid nucleus [55].

mRNA expression of GluN2 subunits was observed increased in CA regions and dentate gyrus in both sclerotic and non-sclerotic TLE patients [48,52]. More recent studies specifically investigating GluN2 subunit subtypes revealed higher expression of mRNA and protein expression of GluN2A and GluN2B subunits in hippocampal resection from patients with mTLE compared to autopsy samples without brain-related disorders and in a TLE pilocarpine model [66]; however, Müller and colleagues found upregulated transcript levels of the GluN2B subunit, but not of the GluN2A subunit, specifically in CA1 pyramidal neurons of TLE rats, suggesting a different distribution of NMDA subunits among hippocampal subfields and suggested a role played by GluN2B in sustaining LTP enhancement at Schaffer collateral-CA1 synapses [67]. By contrast, the analysis of hippocampal and temporal lobe samples collected from chronic TLE animal models showed a reduction in both GluN2A and GluN2B protein concentrations [46,53], although this did not reach statistical power for GluN2B in the hippocampus [53]. No differences in gene expression or receptor densities were found for these subunits in the amygdala of TLE patients compared to specimens obtained postmortem from healthy subjects [55,65]. GluN2A and GluN2B subunits expression seem to be correlated with the incidence of TLE psychiatric comorbidity since the treatment with ifenprodil, a selective antagonist of GluN2B subunits, improved the incidence of depression-like behavior in epileptic pilocarpine-treated rats [68].

Increasing recent studies shed light on micro RNAs (miRNAs) as an effective tool to investigate many brain illnesses. MiRNAs are small RNA molecules that can negatively control their target gene expression post-transcriptionally. In mTLE hippocampus, the reduced expression of the NMDA-GluN1 subunit showed an inverse relationship with miR-219 [54]. In the amygdala of TLE patients compared to healthy human tissues, the significant increase in NMDA-GluN1 subunit correlated with significant down-regulation of miR-219 [54,65], while in the hippocampus of TLE patients, as well as of pilocarpine-treated rodents, a higher expression of mRNA and protein of GluN2A mirrored a down-regulation of miR-139-5p [66].

No data are available for the possible altered expression of the GluN3 subunit in TLE; however, very recent data has shed light on the role of this NMDA receptor subunit in TLE-related neuronal loss as administration of D-serine, an agonist of diheteromeric NMDA receptors, and an antagonist of triheteromeric NMDA receptors, attenuated neuronal loss in the medial entorhinal area and prevented epileptogenesis in pilocarpine-treated rats [69,70].

#### 2.1.3. Kainic Receptors

Among ionotropic glutamatergic receptors, KA receptors are relatively less studied despite the fact that they play a variety of modulatory functions and are peculiarly expressed at both at pre-synaptic and post-synaptic subcellular locations in the majority of principal cells and interneurons in all fields of the hippocampal regions, cortex and amygdala [71,72,73], where recurrent seizures are thought to originate before spreading towards the neocortex. The expression of KA receptors in both GABA-glutamate pre-synaptic boutons can facilitate neurotransmitter release. At the post-synaptic level, KA receptors modulate the synaptic plasticity through the activation of fast excitatory transmission and G-coupled signaling pathway to regulate Ca^2+^-dependent K^+^ conductance, whose activation process remains unknown [74]. KA receptors are tetrameric receptors, with selective permeability for Na^+^ and K^+^ [51], characterized by the combination of five subunits GluK1–GluK5 that co-assemble either forming homomeric (GluK1, GluK2, and GluK3) or heteromeric (GluK4 and GluK5 in association with GluK1–3 subunits) receptors, with peculiar pharmacological and kinetic properties [75]. GluK1 subunits are mostly found in hippocampal interneurons, while GluK2 and GluK5 subunits are situated pre- and post-synaptically in excitatory pyramidal neurons and interneurons [76]. GluK3 is found in dentate gyrus granule cells and in the interneurons of the hippocampal Ammon’s horn, as well as GluK4 that is also present at different expression levels in CA3 and CA1 pyramidal cells and interneurons [77].

KA receptors are largely expressed at presynaptic and postsynaptic sites at mossy fibers-CA3 pyramidal neurons [78]. In TLE tissue, KA influences the sprouting of mossy fibers leading to the generation of recurrent excitatory networks, which are one of the main pathological features in human TLE patients and animal models [79,80]. In addition, the de novo expression of KA receptors in the dentate granule cell layer [81] seems to compromise the perforant path-granule connectivity with strong implications in the normal input-output cortical information processing [82]. Lateral nucleus isolated from the amygdala of TLE patients and compared with amygdala tissues from healthy autopsy tissue display a higher KA receptors density (Table 1), suggesting the possible implication of these receptors in the interictal-like activity along with AMPA receptors [47].

Data obtained from TLE human sufferers reported the increase in GluK1 mRNA and protein levels in the hippocampus, but not in the temporal neocortex, and this upregulation appears to be mainly associated with pre-synaptic facilitation of glutamate release and mossy fibers sprouting that may sustain chronic recurrent seizures [83]. In this regard, GluK1 mRNA was found to be significantly increased in dentate granule cells in mTLE patients compared to both non-sclerotic TLE and hippocampal tissue from autopsies [79]. In the same report, averaged from CA4 to CA2 pyramidal cells showed that GluK1 was unchanged in mTLE patients, but it was reduced in TLE patients without sclerotic hippocampus [79]. GluK2 subunit was found diminished in CA2 and CA4 subfields, but not in the dentate gyrus, in TLE patients with and without sclerotic tissue [79], while no differences resulted from hippocampal and temporal lobe analysis from epileptic rats [46,53]. Furthermore, GluK2-containing KA receptors appeared to be the most involved in the generation of chronic seizures at sprouted mossy fiber synapses [84]. No alterations were reported for the GluK3 subunit in human epileptic tissues [79]. Both GluK4 and GluK5 receptor subunits, located at both pre- and post-synaptic sites, showed elevated expression protein levels in western blot analysis performed in mTLE patients’ hippocampi [51], while specifically in CA2-CA4 areas, Mathern and colleagues found no differences in the mRNA levels compared to healthy postmortem subjects [79]. No modifications in GluK5 receptor level emerged in the same regions in an animal model of TLE [53]. In addition, the GluK5 subunit, but not the GluK4 subunit, was reported to be increased in dentate granule cells of human mTLE [79].

In human and animal models of TLE, the expression of KA receptors is altered in glial cells. In a KA model of TLE, the expression of all KA receptors subunits (GluK1, 2, 4, and 5), in particular, the expression of GluK1 and GluK5 subunits was higher in reactive astrocytes located in the CA1 region eight weeks after SE induction, compared to non-treated animals; however, whether their higher expression ascribes a pro-convulsant or neuroprotective function remains to be clarified [85].

#### 2.1.4. Glutamatergic Metabotropic Receptors

mGluRs actively participate in glutamatergic neurotransmission through the fine control of pre-synaptic neurotransmitter release, also acting as autoreceptors, and post-synaptic neuronal firing. mGluRs are G-protein coupled receptors classified in three groups: Group I (mGluR1 and mGluR5), Group II (mGluR2 and mGluR3), and Group III (mGluR4, mGluR6, mGluR7, and mGluR8) [86].

In the human hippocampus, mGluR1 expression is prominent in CA3 neurons and dentate gyrus. Among the three mGluR1 splice variants (α, β, and d), mGluR1α analyzed in biopsies from healthy subjects was found in interneurons, markedly in the CA1-3 *stratum oriens*, the hilus, and in pyramidal neurons differently distributed among CA fields (CA1–4), but not in glial cells. In these brain areas, as well as in the dentate gyrus and in the basolateral and centromedial nuclei of the amygdala, the mGluR1α distributions were preserved in TLE tissues with both sclerotic and non-sclerotic hippocampi [55,87] and in animal models of TLE [53] (Table 2). By contrast, an up-regulation of mGluR1α subunit was found in the dentate gyrus molecular layer in TLE patients and in an animal model [88], while a reduced amount of protein was found in the temporal lobe samples collected from animal models of TLE [53]. These divergent results found in human TLE were interpreted by Notenboom and colleagues with a mGluR1α antibody cross-reactive with mGluR5 receptors [87].

mGluR5 is expressed at both the pre- and post-synaptic compartments regulating the tripartite synapse in neurons and in astrocytes. Post-synaptically it may elevate intracellular Ca^2+^ concentration through the activation of the phospholipase C pathway [51,89]. Immunohistochemistry and western blot analysis confirmed a strong increase in mGluR5 in hippocampal pyramidal and granule cells, entorhinal cortex, subiculum, and in the dentate gyrus molecular layer in tissues isolated from both sclerotic and non-sclerotic TLE patients [51,87,90]. Significant reduction in mGluR5 was reported in temporal lobe samples from a chronic TLE animal model, but not in the hippocampal region analyzed by Western blot [53]. In addition, recent research findings involving PET-scanner analysis, using the [^11^C]ABP688 tracer, revealed a focal reduction iin mGluR5 binding site in the epileptogenic hippocampal head and amygdala of mTLE patients; this effect seems to be correlated with the severity of the hippocampal impairment since in severe hippocampal atrophy this reduction also involves the ipsilateral temporal neocortex [25]. As suggested by Lam and colleagues, differences observed compared to previous publications [51,87,90] may be related to the different action and sensibility of the PET biomarker binding features; it detects only the allosteric site on the receptors embedded in the plasmalemma, and the binding site may be not available in case of protein conformational changes or internalization, compared to other experimental approaches such as immunohistochemistry and immunoblotting [25].

The elevated expression of excitatory group II receptors, mGluR2 and 3, has mainly been found in the hippocampus, as well as in the amygdala of human TLE samples in comparison with autopsy specimens [47,51] (Table 2). Pre-synaptically, group II mGluRs are coupled to a G-protein that inhibits adenylate cyclase but not Ca^2+^ signaling, suggesting it acts as a counterbalance to prevent hyper-excitation through glutamate clearance from the synaptic cleft [51,91].

Metabotropic glutamatergic receptors belonging to the inhibitory group III, which presynaptically reduced Ca^2+^ influx through the modulation of cyclic AMP levels, were found altered in TLE specimens (Table 2). Compared to control post-mortem biopsies, human mTLE and non-sclerotic TLE samples presented a localized up-regulated immunoreactivity as well as increased transcript levels of mGluR4 subunit in the dentate gyrus and CA4 pyramidal neurons, while in the remaining CA subfields, the mGluR4 expression remained unchanged [92]. Interestingly, presynaptic mGluR8, which regulates glutamate release, was found down-regulated in the components of the lateral perforant pathway using the pilocarpine model [93].

The detection of astrocytic mGluR5 in human and rodent healthy brains [94] is developmentally regulated in the first weeks of life and is downregulated in adulthood. In TLE disease, increased astrocytic expression is seen in adulthood both in humans [51,87,95] and animal model [89], where it plays a role in the glutamate clearance and intracellular Ca^2+^ signaling that in turn regulates astrocytic glutamate release, thus potentially contributing to trigger neuronal excitability [51].

### 2.2. GABAergic Inhibitory Synapses

The ionotropic GABA type A (GABA_A_) and the metabotropic GABA type B receptors (GABA_B_) are the most inhibitory receptors in the brain, mediating fast and slow inhibitory transmission.

GABA transporters (GATs), GAT-1–3, and the betaine/GABA transporter (BGT-1) are expressed in glial cells and pre-synaptic GABAergic neurons and remove the extracellular GABA once its action has been completed, modulating the GABAergic transmission [96]. A comparison between mild and severe sclerotic hippocampi from TLE patients revealed unchanged or locally reduced expression (i.e., hilar region) of neuronal GAT-1 and significantly lower expression of glial GAT-3, suggests a correlation between lower expression and function of GATs with higher degrees of sclerosis [96].

The GABAergic system is actively involved in several pathological conditions of the CNS, including neuronal overactivation that leads to the progression and sustenance of chronic epilepsy and the advent of psychiatric comorbidities such as anxiety and/or depression often associated with TLE [36,97]. In this regard, microdialysis studies in TLE patients’ neocortex revealed a slightly and significant reduction in the GABA content in TLE tissue with and without mood disorders, respectively, in comparison with temporal cerebral tissues isolated from seizure-free patients after cerebral tumor surgery [36].

#### 2.2.1. GABA_A_ Receptors

The specific binding between GABA inhibitory molecules and GABA_A_ ionotropic channels allows chloride (Cl^–^) and bicarbonate (HCO_3_^−^) ions to flow into the cell down their electrochemical gradient, eliciting a hyperpolarizing postsynaptic response [98]. GABA_A_ receptors are heteropentamers of subunits consisting of α1–6, β1–3, γ1–3, δ, ε, θ, π, and ρ1–3 isoforms [99]. Depending on their subunit composition, GABA_A_ receptors differ in terms of affinity for the agonist, kinetic properties, and pharmacological features since they are common targets for antiseizure drugs such as benzodiazepines and barbiturates. Ionotropic GABAergic phasic and tonic neurotransmission modulated by synaptic GABA_A_ receptors are predominantly composed of α1β3γ2 subunits, while extrasynaptic GABA_A_ receptors usually contain δ or ɛ along with α4, α5 or α6 subunits [100].

Evidence from human biopsies and animal models of TLE have demonstrated a variable altered expression and composition of GABA_A_ receptors (Table 3) and heterogeneous allocations among different para-hippocampal areas, including the subiculum, amygdala, and neocortical tissue, in addition to the dentate gyrus and hippocampal region [47,49,55,65,101,102,103,104]. Moreover, the action of GABA signaling in human TLE also appears to be altered due to a reduced affinity of GABA_A_ receptors for the agonist in the hippocampus, temporal neocortex, and especially in the subiculum, increasing the risk of seizure development [105,106].

α1 and α3 subunits appear to be variably reduced in the CA1 and CA2 regions of human mTLE but appear to be unaltered in the hippocampus of TLE tissue, mirroring neuronal loss in these CA subfields [102,103]. By contrast, epileptic rats displayed an increase in the mRNA levels of α1 and α3 subunits in the CA1 region, along with a localized upregulation only for α1 in the CA3 region [101]. No changes for α1 and α3 subunits were observed in the dentate gyrus, hilus, or subiculum [102,103], with the exception of α3 that was found increased in the dentate gyrus and subiculum of TLE tissues [103]. In the KA model of TLE, α1 subunit has been strongly detected in the dentate gyrus along with a reorganization of GABA_A_ receptors subtypes in surviving granule cells, while no changes were observed for both subunits in the subiculum [101]. α1 and α3 subunits were decreased in the entorhinal cortex compared with the autopsy group from healthy controls [104]; in particular, among GABA α subunits, only the α3 subunit was found to be strongly reduced in the superficial layers (I-III) of the temporal neocortex obtained from human mTLE biopsies compared with tissue obtained from healthy patients, using a semiquantitative optical density analysis [107]. Epileptic animals showed α1 subunit upregulation in the perirhinal cortex and entorhinal cortex layer II, without any changes of α3 subunit mRNA expression in either region [101]. Recent studies from human TLE specimens of amygdala reported a bilateral MRI-based volume reduction in TLE patients with hippocampal sclerosis and revealed a global decrease in the expression of α1 and α3 GABA_A_ subunits with internal differences among different amygdala nuclei, in particular in the lateral, basomedial and centromedial complex, compared to autopsies from patients with no brain illnesses [55,104]. Only for basolateral nucleus data are controversial: Jafarian and colleagues found a decrease in α1 receptors, while de Moura et al. and Stefanits et al. obtained with gene expression and immunohistochemistry analysis no differences in the α1 subunit expression [55,65,104]. In the mTLE patient group, a strong upregulation of α2 subunits was detected in the CA2 pyramidal cells layer compared to non-sclerotic tissue and autopsy controls [102]. The expression of the α2 subunit was decreased in the hippocampus and in the lateral, basolateral, and basomedial amygdaloid nuclei [54,104], while it was preserved in the central amygdaloid nucleus and in the temporal neocortex layers, including the entorhinal cortex [54,104,107]. In an animal model of TLE, the gene expression of α2 remained constant 30 and 90 days after SE induction in all brain structures investigated, except in the subiculum, where it appeared to be reduced [101]. Findings obtained from the temporal neocortex sample of TLE patients revealed an increase in α4 subunits expression [36]. An increase in α4 mRNA was reported in KA-model within the dentate gyrus and proximal subiculum, while no changes were reported for the hippocampus, entorhinal, and perirhinal cortices [101]. Finally, no changes in α5 subunit expression were observed in the entorhinal cortex of human mTLE, while general mRNA down-regulation was reported in various amygdaloid nuclei [104], and in a KA model, within the hippocampal, parahippocampal, and temporal cortex regions studied [101].

A general increase in GABA_A_ receptor β1 and β2 subunits have been variably found in human TLE sclerotic and non-sclerotic hippocampal subfields [103]. Within the same study, the β3 immunohistochemical signal was decreased in the CA1 region, increased in the CA2 region, and unchanged in the CA3 region of mTLE tissues compared to healthy controls, while in non-sclerotic specimens, β3 was increased in all hippocampal subregions [103]. More recent reports revealed no differences in β3 gene expression in the hippocampus [54]. Contrary, in experimental animal models, β1 and β2 subunits were found to be unchanged in the CA1 region and reduced in the CA3 region [101], while data about the β3 subunit are divergent; Drexel et al. found no differences [101], while Needs and colleagues found reduced protein levels [53] in hippocampal samples. It is worth noting that Needs and colleagues did not investigate β1 and β2 subunit expression. A general increase in GABA_A_ receptor β1, β2, and β3 subunits was found in TLE with and without hippocampal sclerosis compared to healthy biopsies in the molecular layer of the dentate gyrus, subiculum, and hilus, with the exception of the β3 subunit in the latter [103]. In contrast, in a KA TLE model, these subunits were found almost unchanged in the dentate gyrus and downregulated in the subiculum, entorhinal, and perirhinal cortices [101]. The same GABA_A_ subunit subtypes (β1, β2, and β3) were found to be unchanged in amygdala mTLE samples compared to tissue samples from non-epileptic postmortem specimens [54,65], although a selective reduction in β2 and β3 was found in several human amygdaloid nuclei obtained from mTLE patients [55,104].

In human mTLE samples, γ2 immunoreactivity was reduced in the CA1 region, although no changes were detected in the CA2 or CA3 regions or the hilus [102,103]. However, a recent study found no changes in gene expression of γ2 subunit in the whole mTLE hippocampus compared to autopsy tissues [54]. In the molecular layer of the dentate gyrus and in the subiculum, γ2 immunoreactivity was significantly higher in human mTLE compared to normal hippocampal autopsy cases and to TLE [103]. Interestingly, no immunoreactivity differences emerged in the hippocampal formation, dentate gyrus, subiculum, or hilus in human TLE tissue without hippocampal sclerosis [102,103]. In a rat KA model, no changes were reported in CA1-3 subfields and subiculum, whereas a significant increase was reported in the dentate gyrus [101]. An increase was documented in the γ2 gene expression in the amygdala of TLE patients [54,104] with deep implications for the clustering and kinetics of receptors that are mainly involved in the tonic inhibition [104,107]. In contrast, Jafarian and colleagues [55] reported significantly lower γ2 immunopositivity in the amygdala of mTLE patients, particularly in the basolateral complex and the centromedial nuclei. No changes in γ2 gene expression were found in the temporal cortex [107], including the entorhinal cortex [104,107], while an increase was found in the temporal cortex in a study by Rocha [36]. In a rat KA model, a significant increase was reported in the perirhinal cortex and within the entorhinal cortex layer II [101]. Data from the amygdala and entorhinal cortex are important, considering that about 30% of seizures in TLE are believed to originate in the amygdala and are often associated with emotional disturbances [108]. In this+ regard, other findings obtained from temporal neocortex samples of TLE patients, with or without mood disorders, revealed an increase in γ2 subunits that mediate phasic inhibition at synaptic sites, whereas only the patients without mood disorders showed a concomitant increase in the α4 subunits mRNA that may affect tonic inhibition [36].

The δ subunit participates in tonic inhibition and appeared significantly reduced in the CA1 region, subiculum, entorhinal, and perirhinal cortices, but not in the CA3 or dentate gyrus in epileptic rodents [101].

GABA ionotropic receptors activity is closely dependent, other than on receptor subunit expression, also to the chloride homeostasis. The Na-K-2Cl cotransporter isoform 1 (NKCC1) and the K-Cl cotransporter isoform 2 (KCC2) are the main regulators of intracellular Cl^−^ homeostasis by accumulating and extruding Cl^−^, respectively. Interestingly, chloride cotransporters are expressed differently in males compared to females [109,110]. Brain-specific chloride cotransporters altered expression were found to be implicated in human epilepsy [111,112]; it has been demonstrated, indeed, that it goes along with aberrant GABA excitation contributing to hyperexcitability and possible precipitation of epileptic seizures in TLE [113,114].

ATP has emerged as another key element in GABAergic transmission. During high-frequency neuronal activity, higher rates of ATP release act on low-affinity, slowly desensitizing ionotropic P2X7 receptors, which, in turn, modulates neuronal GABA and glutamate uptake [115]. Increased P2X7 receptor expression in the cortex area of TLE patients down-modulates GABA neurotransmitters, but not glutamate, and increases GABAergic rundown, thus unbalancing the excitation/inhibition system [115].

Very recently, a neural membrane protein known as protrudin has been indicated as relevant in TLE epilepsy; this protein has been implicated in inhibitory signaling through the co-localization with inhibitory synaptic marker gephyrin and regulation of GABA_A_ expression and both phasic and tonic inhibitory function. In particular, immunoblot and immunofluorescence staining have revealed a reduced protein level of protrudin in the temporal neocortex of TLE patients and in the hippocampus and temporal cortex of epileptic mice [116]. Interestingly, the transmembrane protein contactin-associated protein-like 4 (CNTNAP4), which is important in the maturation of inhibitory synapses during development, was lately found reduced in the temporal neocortex of epileptic patients and in both hippocampal and cortical areas of epileptic mice [117]. In particular, the CNTNAP4 protein seems to influence the trafficking of GABA_A_ receptor subunits β2 and β3 through the action of GABA receptor-associated protein affecting inhibitory postsynaptic currents and neuronal excitability [117].

The pathophysiological modulation of GABA_A_ receptors-mediated current is also significantly reduced by cytokines and, in particular, by IL-1β/IL1R1/IRAK1 signaling that is found to be activated in the temporal cortex of TLE patients with or without hippocampal sclerosis, but not in non-epileptic control tissue [118].

#### 2.2.2. GABA_B_ Receptors

Metabotropic GABA_B_ receptors are widely expressed in different regions of the mammalian brain, such as the hippocampus, subiculum, cerebral cortex, and thalamus, and their alteration and involvement in the pharmaco-resistant TLE disorder represent an important subject of study [36,119,120]. GABA_B_ receptors are expressed in excitatory and inhibitory neurons both at pre- and post-synaptic terminals. They are heterodimeric, and pharmacological and physiological studies in both human and animal samples testified the existence of two receptor subtypes, GABA_B1_ (with two isoforms GABA_B1a_ and GABA_B1b_) and GABA_B2_ [119,121]. Pre-synaptic receptor modulates evoked neurotransmitter release regulating Ca^2+^ influx, whereas post-synaptically, it controls neuronal K^+^ current mediating slow and prolonged inhibitory postsynaptic potentials via adenylate cyclase pathway [119].

Sheilabi and colleagues reported a significant increase in GABA_B1_ and GABA_B2_ protein expression in the whole hippocampus, comparing mTLE tissue with autopsy specimens [119] (Table 4). Other studies observed GABA_B2_ receptor expression to be unaltered in the CA2 region and increased in the CA1 and CA3 regions [122], and GABA_B1_ expression was unaltered in the CA2 and CA3 regions and increased specifically in the CA1 region in TLE specimens compared to healthy tissue from autopsy [122,123]. These data were not confirmed in a KA model of TLE, where a hippocampal decrease in mRNA levels and GABA_B_ receptors (GABA_B1_ and GABA_B2_) was observed [120,124]. GABA_B1_ and GABA_B2_ gene expression was observed increased in the hilus and dentate granule cells, while it was unaltered in the subiculum of TLE human tissue compared to autopsy non-epileptic tissue [122,123]. These data were only partially confirmed in animal models of TLE; Straessle et al. reported a decrease in mRNA levels of GABA_B_ receptors in the hilus and an increase in the dentate gyrus, while Furtinger et al. observed no alterations in the latter for both GABA receptor types [120,124]. Recent investigations from mTLE cases compared to control healthy samples revealed a significant reduction in both GABA_B1_ and GABA_B2_ receptor isoforms in the basolateral complex and centromedial amygdaloid nuclei, along with a lower expression of GAD65 enzyme, which catalyzes the conversion of glutamate to GABA [55], while no variations in a GABA_B_ autoradiographic analysis were observed in the lateral amygdala [47].

Electrophysiological recordings and immunostaining for GABA_B_ from layers II/III of human temporal cortical tissue revealed an impairment of GABA_B_-mediated currents possibly due to a lower density of pre- and post-synaptic GABA_B_ receptors expressed in both glutamatergic and GABAergic ending in pharmacoresistant TLE tissue compared to tissues isolated from tumor individuals without pharmacoresistant epilepsy [125]. Interestingly, GABA_B_ receptor levels were then found reduced in all neocortical layers of temporal cortex in TLE patients with anxiety and depression symptoms compared to surgical specimens obtained from non-epileptic patients (brain tumor); this reduction is restricted to neocortical layers V–VI in tissue obtained from TLE patients without psychiatric symptoms [36]. Another example of GABAergic system deficiency is the lacking of crosstalk communication between GABA_A_ and GABA_B_ receptors explored in layer V pyramidal neurons of TLE patients’ cortex, thus contributing to the seizure propagation to subcortical areas [126]; the GABAergic currents were slightly reduced, but not abolished, in terms of presynaptic GABA_B_ autoreceptors, and a decrease in the GABA release might be connected with changes in voltage-gated Ca^2+^ function [126]. The different ability of GABA_B_ receptors to modulate miniature inhibitory postsynaptic current kinetics in TLE tissues may be due to changes in the GABA_B_ receptors expression or post-translational regulation in terms of phosphorylation/dephosphorylation level in pyramidal neurons as well as a different post-synaptic GABA_A_ receptors composition [126].

## 3. Electrical Synapses Features in Temporal Lobe Epilepsy

Electrical gap junctions are a group of hemi-channels that mediate direct intracellular communication between neurons and/or astrocytes, creating an electrically coupled syncytium. Connexon is the structural entity of a gap junction, and it is made up of six-transmembrane proteins called connexins (Cx). The juxtaposition of connexons embedded in the plasma membrane of two neighboring cells forms a hydrophilic pore that promotes the bidirectional propagation of electrical impulses and the transport of small molecules (1–1.2 KDa), such as second messengers and metabolites [127]. The activity of gap junctions is tightly regulated and depends on the number of proteins on the membrane surface, the open-close kinetics of the connexon, and the unitary channel conductance. Hemi-channel gating is also tuned by cytoplasmatic pH and Ca^2+^ concentration [128]. Interestingly, the state of phosphorylation regulates at different stages the intracellular communication between contacting cells, from the connexins gene expression to the channel permeability and the turn-over of the protein expression within the plasma membrane [128,129].

Among the different 20–21 Cxs subunit isoforms found in mammals (21 genes in human and 20 in mice) [130], only nine have been reported in the brain with a region-specific pattern. Mostly Cxs are detected in glial cells (astrocytes and oligodendrocytes) and in a small part of the neuronal population, which included Cx26, Cx32, Cx33, Cx36, Cx37, Cx40, Cx43, Cx45, and Cx46 [131]. In particular, the connexins Cx32, Cx36 and Cx43 are the most represented in the whole CNS. Cx32 is expressed in interneurons and oligodendrocytes, the Cx36 subtype is located in GABAergic interneurons, and Cx43 is largely expressed in astrocytic gap junctions; however, the latter has been sporadically documented between neuron-astrocytes [21,127,132].

In recent years, the involvement of electrical synapses in different neurological disorders, including epilepsy, has attracted increasing attention. Qualitative and quantitative data from biopsy isolated from epileptic and non-epileptic patients has helped shed light on the role of gap junctions in both physiological and pathological conditions.

### 3.1. Astrocytic Gap Junctions

The intracellular communication mediated through gap junctions between astrocytes is mainly accomplished by Cx43 and partly, about 10-fold less, by Cx30 [8]. Gap junctions between astrocytes support a variety of duties, including Ca^2+^ signaling, transport of metabolic compounds, homeostatic clearance, neurotransmitters and K^+^ spatial buffering, tuning neuronal activity, and network synchronization [133,134]. Moreover, they can operate non-channel functions like cell adhesion, migration, and protein interactions [135].

In human mTLE tissue, a strong upregulation of Cx43 was found in the hippocampus, dentate gyrus, subiculum [51,136,137], and in the temporal cortices [138] (Table 5). Higher levels of Cx43 were also discovered in resected epileptogenic foci tissues, including the hippocampus and temporal lobe, removed from patients with different forms of refractory epilepsy, and compared with non-epileptic temporal cortex samples [139]. Higher Cx40 and Cx43 immunofluorescence signals were observed in CA1, CA3, and dentate gyrus of mouse pilocarpine model of TLE [140], while the expression of Cx30 and Cx43 was found unaltered in the hippocampal tissues of pilocarpine, KA, and kindling TLE models [141,142,143].

In human hippocampal sclerotic specimens, total Cx43 levels are higher in comparison with non-sclerotic TLE human biopsies despite a preserved Cx43 levels in the plasma membrane between the two groups, suggesting a dysregulation in the connexins translocation to the plasmalemma surface in sclerotic fraction, whereas no variations were found in the expression of Cx30 in the two groups [8]. Remarkably, the same result concerning the expression of both Cx43 and Cx30 expression was found at the chronic stage of experimental TLE with unilateral-intracortical KA injection compared to the non-injected contralateral hippocampus, which closely recapitulates the results in non-sclerotic TLE samples [8]. Interestingly, data from both sclerotic mTLE human specimens and KA-treated epileptic mice reported that Cx43 is accumulated in large plaques at astrocytic endfeet surrounding blood vessels in the CA1 region [8].

Loss of gap junction coupling was observed in tissue obtained from human mTLE, while gap junctions maintained coupling function in lesion-associated epilepsy without sclerotic or significant morphological alterations in the hippocampal area [144]. This observation seems to be a clinical feature constantly observed in human sclerotic hippocampi regardless of age, gender, or medical treatment of the patients involved in the study [144]. Aberrant astrocytes functions and gap junction uncoupling sustain both an impaired K^+^ buffering capacity [144] and a glutamate uptake deregulation also due to loss of glutamine synthetase has been detected in mTLE, with respect to patients without hippocampal sclerosis and to autopsy controls [145]. Enhanced glutamate and K^+^ levels have been reported in in vivo microdialysis from sclerotic patients; this scenario originates a wave of membrane depolarization that may propagate easily from granule cells to the subiculum [146,147]. The aberrant expression of Cx43 may lead to a deregulation of the direct cross-talk communication within the astrocytic syncytium, supporting the onset of synchronized seizures and leading the progression from a partial seizure to a generalized one even involving areas far from the site where seizures are originated [137]. In primary rat mixed cultures of astrocytes prepared from rat cerebral cortices, this process seems to be related to an increase and rapid Ca^2+^ waves propagation between coupled astrocytes or to other cells, due to a glutamate overload in the synaptic cleft that also results in both neuro- and glio-toxic effects [148]. Interestingly, patch-clamping experiments in human drug-resistant TLE specimens have revealed a more depolarized astrocytic resting membrane potential compared to cortical astrocytes [149], along with astrocytes being resected from the epileptic foci displaying higher sodium channels densities, adapted to generate action potential-like responses [149].

The phosphorylation status of the Cx43 subunits is a key element to control the gap junction kinetics. Among others, specific phosphorylations at the C-terminal tail at positions S255 and S368, targeted by mitogen-activated protein kinase (MAPK) and protein kinase C (PKC), respectively, have shown reduced gap junctions open probability and the electric current passing through the junction [150,151,152]. In that regard, enhanced phosphorylation was found specifically at position S255 rather than S368 in human specimens [144], whereas in a KA acid experimental TLE model, the enhanced post-translational phosphorylation was observed in both sites [8]. It is worth noting that the state of phosphorylation in chronic human brain biopsy may be affected by other conditions, such as medical treatment that could explain the different results in both humans and in animal TLE models [8]. In addition, the data in the literature postulate other potential phosphorylation sites involved in the human sclerotic brain, but S255 seems to be the main one so far [8].

Neuroinflammation has been reported to play a pivotal role in astrocytic coupling both in cell culture and in human specimens [132,153]. Epileptic seizures alter blood-brain barrier permeability leading to albumin and other serum proteins extravasation into the brain parenchyma [154,155,156] that in turn activates the transforming growth factor beta (TGFβ) receptor-mediated signaling cascade in astrocytes [157]. The TGFβ pathway mediates the activation of MAPKs that can directly and/or indirectly (i.e., different gene expression) modulate the Cx43 phosphorylation level. Analyses of serum and cerebrospinal fluid of epileptic patients have confirmed higher levels of cytokines such as IL-1B and tumor necrosis factor (TNF) [8,146,153].

### 3.2. Neuronal Gap Junctions

Although gap junctions have been more studied in glial cells, their functions and involvement in TLE disorder have also been described in neuronal cells. Among the different Cx subtypes sustaining the communication between neurons, Cx36 emerges as the major isoform expressed since the evidence of almost 95% neuronal uncoupling in Cx36 knockout mice [158]. Cx36 subtype has been found in the mammalian neurons of different brain areas, including granule cells in the dentate gyrus (mossy fibers) and hippocampus, mainly localized in the CA3 and CA4 subfields [136,159], and it is mostly expressed in GABAergic interneurons [142].

Human sub-temporal amygdalohippocampectomy resections from TLE patients have revealed a Cx36 expression comparable to non-epileptic patients in all CA sub-fields and the dentate gyrus and subiculum [136]. Major data about the role of Cx36 in the TLE come from experiments in animal epilepsy models and sometimes appear controversial. Unaltered Cx36 expression has been observed in hippocampal tissue obtained from KA [143], pilocarpine [142], and kindling [141,143] animal models of TLE. On the contrary, recent investigations in pilocarpine-treated epileptic mice indicated a significant decrease in the gene expression and immunohistochemistry analysis of Cx36 in the CA1 and CA3 regions and the dentate gyrus, whereas a localized increase in Cx36 was found in the *stratum lucidum* of the CA3 region, favoring a tight connection between mossy fibers and CA3 neurons supporting neuronal hyperexcitability [159].

Cx32 subtypes have been found between neuronal subpopulations as well as in oligodendrocytes [160] and sustain K^+^ buffering and the movement of nutrients and ions [127,161]. Collignon and colleagues showed a general reduction in the expression level of Cx32 in human TLE compared with non-epileptic control in the *stratum pyramidale* of different hippocampal CA subregions and in the subiculum, but not in the dentate gyrus, although the discrimination of the expression level between the neuronal and oligodendrocyte cell populations was not specified [136]. On the contrary, upregulation of Cx32 was found in the epileptogenic foci, including the hippocampus and temporal lobe, obtained from patients with various medically refractory epilepsies, including TLE, compared to non-epileptic subjects used as controls [139]. In human samples from the epileptic temporal lobe neocortex, levels of Cx32 mRNA were similar to levels observed in samples from the non-epileptic peritumoral temporal neocortex [138]. Significant alterations in Cx32 mRNA expression have not been observed in the hippocampus of kindling or KA animal TLE models [143].

## 4. Discussion and Conclusions

TLE, with or without mesial temporal sclerosis, is one of the most frequent forms of focal epilepsy, often characterized by a strong resistance to common AEDs. Since the etiology of TLE is very difficult to identify, it is commonly associated with traumatic brain episodes that trigger the activation of several precipitating events altering, among others, synaptic homeostasis and finalizing the generation of spontaneous convulsive seizures. This review centers the attention on qualitative and quantitative chemical and electrical synaptic alterations which characterize different limbic areas in human and animal models of TLE.

AMPA receptor subunits are found generally increased in the hippocampus and dentate gyrus of human TLE, although these results are often not confirmed in the animal models where a decreased expression is sometimes reported. Among the metabotropic glutamate receptor subunits, mGluR5 is increased in the hippocampus and dentate gyrus in human TLE, while an increase in mGluR4 is limited to the CA4 subfield. GABA type A receptor subunits are found mainly unchanged or increased in the dentate gyrus, hilus, and subiculum of human TLE, while no consensus has been reached on the hippocampal expression where big differences have been observed comparing subunit types both in human and animal TLE tissue. The divergence of data is more limited when referring to GABA type B receptor subunits, found increased in the hippocampus, dentate gyrus, and hilus in human TLE, but has again not been confirmed in animal models. A general increase in glutamatergic receptors, along with a general decrease in GABAergic ionotropic receptors and an increase in gap junction subunits causes an unbalance between excitation and inhibition. In some areas, it has been shown an increase in GABA receptor subunits when compared to healthy conditions suggesting an attempt to counteract, without success, hyperexcitability of the neuronal network [120,124].

The studies mentioned in this review may present some technical limitations related to the preservation of the tissue quality after isolation [162]. It has been demonstrated in both human and rodents that the postmortem delay and the temperature conditions after surgery may differently affect the expression of protein [162] and mRNA content [163] in a time-dependent manner, thereby increasing the risk of collecting inaccurate data; this may explain the experimental variations found among different studies. In addition, it has been demonstrated that some proteins are more stable than others. NMDA receptors NR2A and NR2B subunits, for example, have been reported to be more liable to degradation in respect to other ionotropic glutamatergic subunits [164].

Current knowledge about the neurobiology of epilepsy in humans mainly emerges from autopsies, from surgical resections obtained from patients with pharmacoresistant TLE who underwent temporal lobectomy [22], or from living subjects using PET. In all cases, sampling bias due to the selection of subjects that may not be representative of the whole spectrum of TLE scenarios can create limitations for the study of TLE using human tissue. Surgical specimens are obtained from TLE/mTLE patients presenting pharmacoresistant seizures, and the evidence of a unique operable epileptic focus has been proved by preliminary imaging and electroclinical data [8,51,83,119,122]. For PET investigations, in addition to adulthood age and legal capacity to give informed consent, general inclusion criteria may comprise refractory focal epilepsy (among which, but not exclusively, mTLE), patients eligible for surgical resection, and absence of previous ipsilateral cerebral surgery. In addition, experimental groups likely include patients under different drug treatments at the time of the study (monotherapy or polytherapy with different AEDs) or who had tried in the past various drug therapies, increasing the inhomogeneity of the sample both in the postsurgical specimen or in vivo studies.

In human studies, especially those involving surgical specimens, the control group often includes surgical resection obtained from non-epileptic people affected by other pathologies, such as glioma. The introduction of less invasive techniques, such as PET, is of great help as it can be performed on healthy volunteer subjects [25,26,27]. However, although control groups may consist of healthy subjects often selected by stringent requirements, they may not be homogeneous with respect to the experimental group distribution in relation to sample size, demographic (age and gender), geographic, and clinical characteristics (i.e., body mass index and no sign of neither neurological nor psychiatric disorders) [165]. In addition, there is not always a guarantee that control subjects are not taking medications for non-epileptic pathologies as well; therefore, it may not be homogeneous in this sense either [25,26,27].

Intra-subject and inter-subject differences represent another limitation of the reported studies. Inter-hemispheric differences within the same subject are never taken into consideration, both in human and rodent studies, although it has been demonstrated that brain anatomical features and connectivity in the left and right hemispheres differently affect the extension of the lesion and seizures propagations on adjacent brain structures [10,166]. In addition, none of the studies mentioned in this review reported data about male versus female subjects, although anatomical sexual dimorphism that can influence seizure susceptibility and neurodegeneration processes has been reported [166,167]. Investigations regarding gender disparity in human mTLE patients revealed anatomical damage differences between men and women with the major involvement of frontal regions in men and temporal areas in women, in addition to large differences in membrane protein expression, transcription factors activation that in turn may influence the clinical manifestation of epileptic seizures [109,110,166]. In this regard, gender medicine and precision medicine have been recently attracting a lot of attention from scientists. Sex and gender are increasingly recognized as major influencing factors in all disorders, including brain diseases. Gender and sex have emerged as the potential drivers of patient diversity in terms of disease onset, progression, diagnosis, and treatment, and implementing guidelines is becoming prevalent in most areas of medicine [168]. However, many basic and preclinical studies, including those on TLE animal models reported in this review, have been conducted mostly on male cohorts. More accurate data analysis on the expression of subunit/receptor involved in chemical and electrical synapses in women versus men might lead to more successful clinical outcomes in TLE as in many other brain disorders [169].

Take together, these results provide evidence for the complexity that thoroughly regulates the synaptic function and how much is still unknown; therefore, future experiments will be needed to shed light on many unanswered questions. Modern imaging techniques represent a non-invasive investigation alternative which, together with the evolution of innovative biomarkers with ever-higher specificity for the different receptor subunits, are promising tools to refine and render our knowledge about synaptic alterations in TLE more accurately.

## Figures and Tables

**Table 1:** Alterations of neuronal glutamatergic ionotropic receptors/subunits.

	Hippocampus	DG	Amg	Temp ctx	TLE Model	Refs
**AMPA**				↑	**Human**	[27]
			**↑(La)**		**Human**	[47]
				↑	**Human**	[49]
**GluA1**			=(BLA and CeM)		Human	[55]
	↑				Human	[51]
	↑/↑	↑/↑			Human	[48]
	↑/↑	↑/↑			Human	[52]
		↑			Human and Rat KA	[50]
	↓				Rat PILO	[53]
	↓				Rat KA	[46]
**GluA2**	=		↑		Human	[54]
			=(BLA and CeM)		Human	[55]
	↓				Human	[51]
	↑/↑	↑/↑			Human	[48]
	=/↑	↑/↑			Human	[52]
	↓				Rat PILO	[53]
	↓				Rat KA	[46]
**GluA3**	↑/↑	↑/↑			Human	[48]
	=/=	=/=			Human	[52]
	↓				Rat PILO	[53]
**NMDA**			**=(La)**		**Human**	[47]
		↑			**Human**	[64]
**GluN1**	↓		↑		Human	[54]
			=(BLA and CeM)		Human	[55]
			↑		Human	[65]
	↑/↑	↑/↑			Human	[48]
	=/=	=/↑			Human	[52]
	=				Rat PILO	[53]
**GluN2A**			=(BLA)		Human	[65]
	↑				Human and Rat PILO	[66]
	↓				Rat PILO	[53]
	=(CA1)				Rat PILO	[67]
**GluN2B**			=(BLA and CeM)		Human	[55]
			=(BLA)		Human	[65]
	↑				Human and Rat PILO	[66]
	↑/↑	↑/↑			Human	[48]
	=				Rat PILO	[53]
	↓				Rat KA	[46]
	↑(CA1)				Rat PILO	[67]
**KA**			**↑(La)**		**Human**	[47]
**GluK1**	↑			=	Human	[83]
	↓(CA2–4)/=	=/↑			Human	[79]
**GluK2**	↓(CA2-4)/↓(CA2-4)	=/=			Human	[79]
	=				Rat PILO	[53]
	=				Rat KA	[46]
**GluK3**	=/=	=/=			Human	[79]
**GluK4**	↑				Human	[51]
	=/=	=/=			Human	[79]
**GluK5**	↑				Human	[51]
	=/=	=/↑			Human	[79]
	=				Rat PILO	[53]

Upregulation (↑), downregulation (↓), or no changes (=) of glutamatergic ionotropic receptor (grey background)/subunit (no highlighting) expression in TLE (without TLE/mTLE specifications), or TLE/mTLE when separate data are available, compared to non-epileptic controls. Amg: Amygdala; BLA: Basolateral amygdaloid nucleus; CeM: Central amygdaloid nucleus, medial; DG: Dentate Gyrus; KA: Kainic acid; La: Lateral amygdaloid nuclei; PILO: Pilocarpine; Refs: References; Temp ctx: Temporal neocortex.

**Table 2:** Alterations of neuronal glutamatergic metabotropic receptors.

	Hippocampus	DG	Sub	Amg	Temp ctx	TLE Model	Refs
**Group I**							
**mGluR1α**				=(BLA and CeM)		Human	[55]
	=/=	=/=				Human	[87]
		↑				Human, Rat KA, and Rat Kindling	[88]
	=					Rat PILO	[53]
**mGluR5**	↓(head)			↓	↓	Human	[25]
	↑	↑	↑		↑(Ent)	Human	[90]
	↑					Human	[51]
	↑/↑	↑/↑				Human	[87]
	=					Rat PILO	[53]
**Group II**							
**mGluR2/3**	↑					Human	[51]
				↑(La)		Human	[47]
**Group III**							
**mGluR4**	↑(CA4) =(CA1–3)/↑(CA4) =(CA1–2–3)	↑/↑				Human	[92]
**mGluR8**	↓					Rat PILO	[93]

Upregulation (↑), downregulation (↓), or no changes (=) of glutamatergic metabotropic receptor expression in TLE (without TLE/mTLE specifications), or TLE/mTLE when separate data are available, compared to non-epileptic controls. Grey background highlights the metabotropic receptors main groups. Amg: Amygdala; BLA: Basolateral amygdaloid nucleus; CeM: Central amygdaloid nucleus; DG: Dentate Gyrus; Ent: Entorhinal cortex; KA: Kainic acid; PILO: Pilocarpine; Refs: References; Sub: Subiculum; Temp ctx: Temporal neocortex.

**Table 3:** Alterations of neuronal GABAergic ionotropic receptors/subunits.

	Hippocampus	DG	Hilus	Sub	Amg	Temp ctx	TLE Model	Refs
**GABA_A_**					**↓(La)**		**Human**	[47]
**α1**					↓(BLA and CeM)		Human	[55]
					=(BLA) ↓(La, BM, and CeM)	↓(Ent)	Human	[104]
					=(BLA)		Human	[65]
						=/=	Human	[107]
	=/↓(CA1) =(CA2–3)	=/=	=/↓	=/=			Human	[103]
	=(CA2)/↓(CA2)	=/↑					Human	[102]
	↑(CA1-3)	↑		=		↑(Ent Lyr II and Per)	Rat KA	[101]
**α2**	↓				=		Human	[54]
					↓(La, BLA and BM) =(CeM)	=(Ent)	Human	[104]
						=	Human	[107]
	=(CA2)/↑(CA2)	=/↑					Human	[102]
	=(CA1–3)	=		↓		=(Ent and Per)	Rat KA	[101]
**α3**					↓	↓(Ent)	Human	[104]
						↓(Lyr I–III)/↓(Lyr I–III)	Human	[107]
	=/↓(CA1) =(CA2–3)	↑/=	=/=	↑/=			Human	[103]
	=(CA2)/↓(CA2)	=/=					Human	[102]
	↑(CA1) =(CA3)	=		=		=(Ent and Per)	Rat KA	[101]
**α4**						↑	Human	[36]
	=(CA1–3)	↑		↑(Proximal)		=(Ent and Per)	Rat KA	[101]
**α5**					↓(La, BLA, BM, and CeM)	=(Ent)	Human	[104]
	↓(CA1–3)	↓		↓		↓(Ent and Per)	Rat KA	[101]
**β1**					=(BLA)		Human	[65]
	↑(CA3) =(CA1–2)/↑(CA2) =(CA1–3)	↑/↑	↑/↑	↑/↑			Human	[103]
	↓(CA3) =(CA1)	=		↓(Distal)		↓(Ent Lyr V–VI and Per)	Rat KA	[101]
**β2**					↓(La) ↑(CeM) =(BLA and BM)	↓(Ent)	Human	[104]
					=(BLA)		Human	[65]
	↑(CA1–3) =(CA2)/↑(CA1–2–3)	↑/↑	=/↑	↑/↑			Human	[103]
	↓(CA3) =(CA1)	=		↓		↓(Ent Lyr V–VI and Per)	Rat KA	[101]
**β3**	=				=		Human	[54]
					↓(BLA and CeM)		Human	[55]
	↑(CA1–2–3)/↓(CA1)↑(CA2) =(CA3)	↑/↑	=/=	↑/↑			Human	[103]
	↓						Rat PILO	[53]
	=(CA1–3)	=		↓		↓(Ent and Per)	Rat KA	[101]
**γ2**	=				↑		Human	[54]
					↓(BLA and CeM)		Human	[55]
					↑	=(Ent)	Human	[104]
						↑	Human	[36]
						=/=	Human	[107]
	=/↓(CA1) =(CA2–3)	=/↑	=/=	=/↑			Human	[103]
**γ2**	=(CA2)/=(CA2)	=/↑					Human	[102]
	=(CA1–3)	↑		=		↑(Ent Lyr II and Per)	Rat KA	[101]
**δ**	↓(CA1) =(CA3)	=		↓		↓(Ent and Per)	Rat KA	[101]

Upregulation (↑), downregulation (↓), or no changes (=) of γ-aminobutyric acid (GABA)ergic ionotropic receptor (grey background)/subunit (no highlighting) expression in TLE (without TLE/mTLE specifications), or TLE/mTLE when separate data are available, compared to non-epileptic controls. Amg: Amygdala; BLA: Basolateral amygdaloid nucleus; BM: Basomedial amygdaloid nuclei; CeM: Central amygdaloid nucleus; DG: Dentate Gyrus; Ent: Entorhinal cortex; KA: Kainic acid; La: Lateral amygdala; Lyr: Layer; Per: Perirhinal cortex; PILO: Pilocarpine; Refs: References; Sub: Subiculum; Temp ctx: Temporal neocortex.

**Table 4:** Alterations of neuronal GABAergic metabotropic receptors.

	Hippocampus	DG	Hilus	Sub	Amg	Temp ctx	TLE Model	Refs
**GABA_B_**						**↓**	**Human**	[36]
					**=(La)**		**Human**	[47]
						**↓**	**Human**	[125]
**GABA_B1_**					↓(BLA and CeM)		Human	[55]
	↑						Human	[119]
	↑(CA1) =(CA2–3)	↑	↑	=			Human	[122]
	↑(CA1) =(CA2–3)	↑	↑				Human	[123]
	↓(CA1–2–3)	=					Rat KA	[124]
	↓(CA1–3)	↑	↓				Mouse KA	[120]
**GABA_B2_**					↓(BLA and CeM)		Human	[55]
	↑						Human	[119]
	↑(CA1–3) =(CA2)	↑	↑	=			Human	[122]
	↓(CA1–2–3)	=					Rat KA	[124]
	↓(CA1–3)	↑	↓				Mouse KA	[120]

Upregulation (↑), downregulation (↓), or no changes (=) of GABAergic metabotropic receptor expression in TLE (without TLE/mTLE specifications) compared to non-epileptic controls. Grey background highlights the metabotropic GABA_B_ receptors main group. Amg: Amygdala; BLA: Basolateral amygdaloid nucleus; CeM: Central amygdaloid nucleus; DG: Dentate Gyrus; KA: Kainic acid; La: Lateral amygdaloid nuclei; Refs: References; Sub: Subiculum; Temp ctx: Temporal neocortex.

**Table 5:** Alterations of gap junction subunits.

	Hippocampus	DG	Sub	Temp ctx	TLE Model	Refs
**Cx30**	=				Rat KA, Rat Kindling	[143]
**Cx32**	↑			↑	Human	[139]
	↓	=	↓		Human	[136]
				=	Human	[138]
	=				Rat KA and Rat Kindling	[143]
**Cx36**	=	=	=		Human	[136]
	↓(CA1–3)	↓			Mouse PILO	[159]
	=				Rat PILO	[142]
	=				Rat Kindling	[141]
	=				Rat KA and Rat Kindling	[143]
**Cx40**	↑(CA1–3)	↑			Mouse PILO	[140]
**Cx43**	↑			↑	Human	[139]
	↑				Human	[51]
	↑	↑	↑		Human	[136]
	↑(CA1–4)				Human	[137]
				↑	Human	[138]
	=				Rat PILO	[142]
	↑(CA1–3)	↑			Mouse PILO	[140]
	=				Rat Kindling	[141]
	=				Rat KA and Rat Kindling	[143]

Upregulation (↑), downregulation (↓), or no changes (=) of gap junction subunit expression in TLE (without TLE/mTLE specifications) compared to non-epileptic controls. DG: Dentate Gyrus; KA: Kainic acid; PILO: Pilocarpine; Refs: References; Sub: Subiculum; Temp ctx: Temporal neocortex.

## Abbreviations

AED	Antiepileptic drug
AMPA	α-Amino-3-hydroxy-5-methylisoxazole-4-propionic acid
CA	*Cornu Ammonis*
CNS	Central nervous system
CNTNAP4	Protein contactin-associated protein-like 4
Cx	Connexin
GABA	γ-Aminobutyric acid
GAT	GABA transporter
KA	Kainic acid
LTP	Long term potentiation
MAPK	Mitogen-activated protein kinase
mGluR	Metabotropic glutamate receptor
miRNA	Micro RNA
MRI	Magnetic resonance imaging
mTLE	Mesial temporal lobe epilepsy
NMDA	N-methyl-D-aspartate
PET	Positron emission tomography
SE	*Status epilepticus*
SRS	Spontaneous recurrent seizure
TLE	Temporal lobe epilepsy
